# 959 Retrospective Descriptive Study of Scalp Burns at Urban Level 1 Pediatric Trauma Center

**DOI:** 10.1093/jbcr/iraf019.490

**Published:** 2025-04-01

**Authors:** Sara Ma, Carolyn Baldwin, Christina Shanti, Mihaela-Elena Rapolti

**Affiliations:** Wayne State University School of Medicine; Wayne State University School of Medicine; Department of Surgery, Division of Pediatric Surgery, Children’s Hospital of Michigan, Wayne State University; Michael & Marian Ilitch Department of Surgery, Wayne State University School of Medicine, Detroit Medical Center

## Abstract

**Introduction:**

Pediatric burns account for a significant number of injuries reported to emergency departments nationwide. Although scalp burns are relatively uncommon, they carry high risk for long-term disfigurement and increased psychosocial burned. Management can be complex, and reconstruction of post-burn alopecia is an option in select patients. Timely, definitive care is therefore critical to minimize patient morbidity and optimize long-term, aesthetic outcomes. Therefore, we conducted a descriptive study to understand patient demographics and resource allocation at our high-volume urban Level 1 pediatric trauma center to understand and improve scalp burn outcomes.

**Methods:**

This study is a retrospective analysis of pediatric patient scalp burns, as identified with ICD-10 codes. All relevant patient records from our institution between January 2016 to February 2024 were analyzed using descriptive statistics.

**Results:**

During this 8-year 2-month period, we identified 4,791 pediatric patients with burn injuries that were treated at our institution. Of these, 175 patients (3.65%) suffered from scalp burns. Our patient population encompassed 112 African American, 51 Caucasian, 2 Asian, and 7 other ethnicity patients. Primary etiologies were scald (59.4%), flame (19.4%), and friction (15.4%). Overall, we identified 170 reported cases of second-degree burns (total body surface area TBSA 5.9% ± 6.9) and 23 reported cases of third-degree burns (TBSA 12.7% ± 17.9). The majority of scalp burn patients (51%) were brought from an outside referring facility. On average, length of hospitalization was 7.4 days, with 21 readmissions (12%). Thirteen patients with scalp burns (7.4%) had the diagnostic of 3rd degree burn. Seven patients of them (53.4%) needed skin graft and the rest of them healed by secondary intention. Only 2 patients with skin grafts (15.4%) had reconstruction of their postburn alopecia.

**Conclusions:**

Our study reports an unexpectedly low proportion of pediatric reconstruction for post-traumatic scalp burn alopecia. High-fidelity regeneration of injured scalp tissue, relatively small scar size, or ease of scar concealment at the hairline or with long hair reduce desire for reconstruction. A patient’s family’s lack of knowledge of reconstructive options and perceived lack of surgical necessity would also significantly affect reconstruction efforts. Without timely reconstruction, however, pediatric scalp burns can result in long-term psychological burden. Education of patient families and inclusion of plastic surgeons on a multi-disciplinary burn team could therefore improve long-term patient outcomes.

**Applicability of Research to Practice:**

Elucidation of patient demographics, burn etiologies, and care management can shape resource allocation and reduce barriers to receiving proficient longitudinal burn care for pediatric scalp injuries.

**Funding for the Study:**

The authors received no external funding for this study.

